# Re-evaluation of the origin of relaxor ferroelectricity in vinylidene fluoride terpolymers: An approach using switching current measurements

**DOI:** 10.1038/s41598-017-16017-w

**Published:** 2017-11-20

**Authors:** Naoto Tsutsumi, Kyohei Okumachi, Kenji Kinashi, Wataru Sakai

**Affiliations:** 1 Faculty of Materials Science and Engineering, Kyoto Institute of Technology, Matsugasaki, Sakyo, Kyoto, 606-8585 Japan; 2 Master’s Program of Innovative Materials, Graduate School of Science and Technology, Kyoto Institute of Technology, Matsugasaki, Sakyo, Kyoto, 606-8585 Japan

## Abstract

Relaxor-ferroelectric vinylidene fluoride (VDF)-based terpolymers have attracted increased attention for industrial applications because of their large dielectric constants, low voltage operation for nonvolatile memory, and energy storage capabilities. However, the origin of the relaxor ferroelectricity of VDF-based terpolymers is still under investigation. Here, we investigate the ferroelectric behaviour of thin films of terpolymers of VDF, trifluoroethylene (TrFE), and chlorofluoroethylene (CFE) (P(VDF-TrFE-CFE)) and terpolymers of VDF, TrFE, and chlorotrifluoroethylene (CTFE) (P(VDF-TrFE-CTFE)) using switching current – electric field (I-E) loop measurements. I-E loop measurements have substantial advantages because they directly provide information regarding the independent switching behaviour of dipoles. We show that the I-E loops of P(VDF-TrFE-CFE) are the summation of three pairs of Gaussian peak functions. Moreover, we provide definite proof of the presence of double hysteresis loop-like antiferroelectric behaviour and relaxor-ferroelectricity in the nanodomains of the dipoles when applying positive or negative sinusoidal electric fields to the sample films.

## Introduction

Since the piezoelectric response of poly(vinylidene fluoride) (PVDF) was first reported in 1969^1^, followed by a report on the pyroelectricity of PVDF in 1971^2^, vinylidene fluoride (VDF)-based fluoropolymers have attracted increased attention in the communities of polymer physics, solid-state physics, and device development. The piezoelectricity and pyroelectricity in PVDF originate from the polar crystal dipoles of the β-form, δ-form, and γ-form^3,4^. The switching reversal of these polar crystals while applying alternative external electric fields leads to ferroelectricity in PVDF. The addition of trifluoroethylene (TrFE) comonomers successfully enables the preferential formation of β-crystal forms, which possess the largest net dipoles. The wider lattice spacing introduced by the bulky TrFE comonomers allows the dipoles to rotate more easily in the thin film while applying an alternative electric field.

Stable bipolar states in ferroelectric polymers are promising for use in the fields of thin film nonvolatile memory devices and printed electronics for commercial use^5^. Thin film technology has also enabled fast dipole switching in copolymers of VDF and TrFE (P(VDF-TrFE))^6–8^.

The further addition of a third comonomer such as chlorofluoroethylene (CFE) or chlorotrifluoroethylene (CTFE) leads to terpolymers of VDF, TrFE, and CFE (P(VDF-TrFE-CFE)) and terpolymers of VDF, TrFE, and CTFE (P(VDF-TrFE-CTFE)), which are so-called relaxor ferroelectrics^9,10^. They have many promising features, such as high dielectric constants (high k values), low dielectric losses with their broad dispersion against temperature for applications in piezoelectric devices and condensers, and energy storage^11^ and electrocaloric capabilities^12^ for industrial applications. In organic thin film transistors (OTFT), relaxor-ferroelectric terpolymers are suitable for high-k gate materials^13^. Thin films of P(VDF-TrFE-CFE) terpolymers have been used as nonvolatile memory devices with a low-voltage operation of 1 V for integrated drive electronics^14^.

Physical pinning against ferroelectric switching due to heavy chlorine atoms has been proposed to explain the relaxor-ferroelectric behaviour in such terpolymers^10^. However, the characteristics of the switching mechanism due to physical pinning in those terpolymers is still under investigation and discussion. Commonly and widely used methods include displacement – electric field (D-E) hysteresis loop or polarization – electric field (P-E) hysteresis loop measurements. In contrast, our methodological approach involves current – electric field (I-E) loop measurements. I-E loops have substantial advantages because they directly provide information regarding independent polarization reversals. The D-E or P-E loops can be computed by integrating the I-E loop.

Here, we investigate the origin of relaxor ferroelectricity in P(VDF-TrFE-DFE) terpolymers using I-E loop measurements. Using a peak separation technique, the I-E loop was reproduced using three independent Gaussian peaks for the relaxor and double hysteresis components. A definite measurement of the double hysteresis loop is also demonstrated by only applying a positive or negative electric field.

## Results and Discussion

The Curie temperatures and melting points were measured using DSC and are listed in Table 1. The Curie temperatures of P(VDF-TrFE-CFE) and P(VDF-TrFE-CTFE) were within the vicinity of room temperature. The lattice spacings were calculated using Bragg’s equation. The introduction of bulky CFE or CTFE comonomer sites led to wider lattice spacings, as shown in Table 1. The relative dielectric constants were 45–55 for P(VDF-TrFE-CFE), 29–31 for P(VDF-TrFE-CTFE), and 9–11 for P(VDF-TrFE), which were calculated from the switching current analysis.

**Table 1:** Curie temperature (T_c_), melting point (T_m_), peak 2θ, and lattice spacing for each ferroelectric polymer.

Sample	T_c_ (°C)	T_m_ (°C)	Peak 2θ (°)	Lattice spacing (nm)
P(VDF-TrFE-CFE)	19.4	127.7	18.2	0.49
P(VDF-TrFE-CTFE)	23.3	120.6	18.3	0.48
P(VDF-TrFE)	119.5	149.8	19.9	0.45

T_c_ and T_m_ were measured using DSC. Peak 2θ was measured using WAXD. The lattice spacing was calculated using Bragg’s equation.

**Figure 1:**
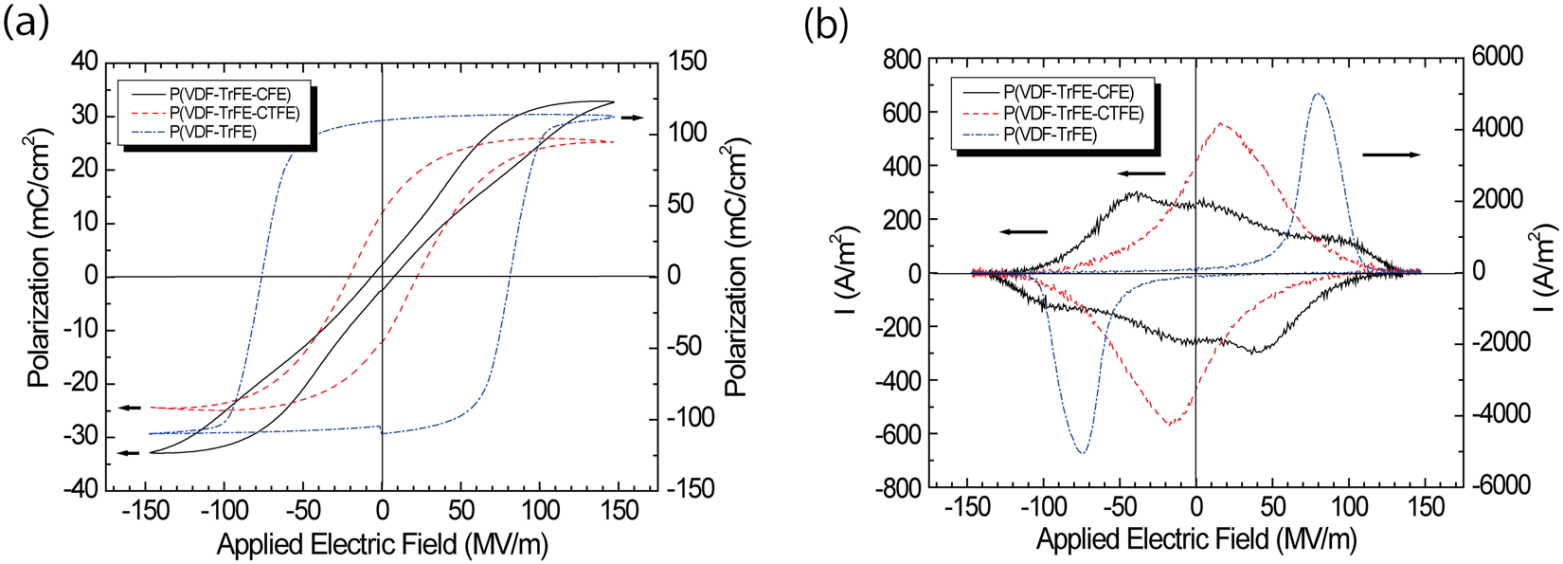
(**a**) P-E hysteresis loop, (**b**) corresponding I-E loop when a sinusoidal electric field with a 1k Hz cycle was applied to the sample film. Black solid line: P(VDF-TrFE-CFE) terpolymer, red dashed line: P(VDF-TrFE-CTFE) terpolymer, and blue dashed dotted line: P(VDF-TrFE) copolymer. In both figures, the left vertical axis is for the P(VDF-TrFE-CFE) and P(VDF-TrFE-CTFE) terpolymers, and the right vertical axis is for P(VDF-TrFE). The top gold electrode area was 117 μm × 117 μm. Thickness: 223.2 nm for P(VDF-TrFE-CFE), 223.9 nm for P(VDF-TrFE-CTFE), and 205 nm for P(VDF-TrFE).

Displacement – electric field (D-E) hysteresis or polarization – electric field (P-E) hysteresis has commonly been used to evaluate ferroelectricity due to spontaneous dipoles switching by applying an external electric field to vinylidene fluoride polymers. Typical P-E hysteresis curves for P(VDF-TrFE-CFE), P(VDF-TrFE-CTFE), and P(VDF-TrFE) are shown in Fig. 1(a). The hysteresis curve of P(VDF-TrFE-CFE) exhibits relaxor- and antiferroelectric-like responses with a very small coercive field^10^, whereas P(VDF-TrFE) exhibits a distinct and large coercive field (E_c_) and large remanent polarization (P_r_)^8^. The corresponding loop responses in the current – electric field (I-E loop) are shown in Fig. 1(b). The P(VDF-TrFE-CFE) terpolymer exhibits a complex switching current loop as a function of the applied electric field. In contrast, the P(VDF-TrFE) copolymer shows a simple reversal response due to cooperative switching of the VDF-TrFE dipoles^8^. The complex switching current response in P(VDF-TrFE-CFE) notably differed from the well-known simple switching reversal behaviour of P(VDF-TrFE).

**Figure 2:**
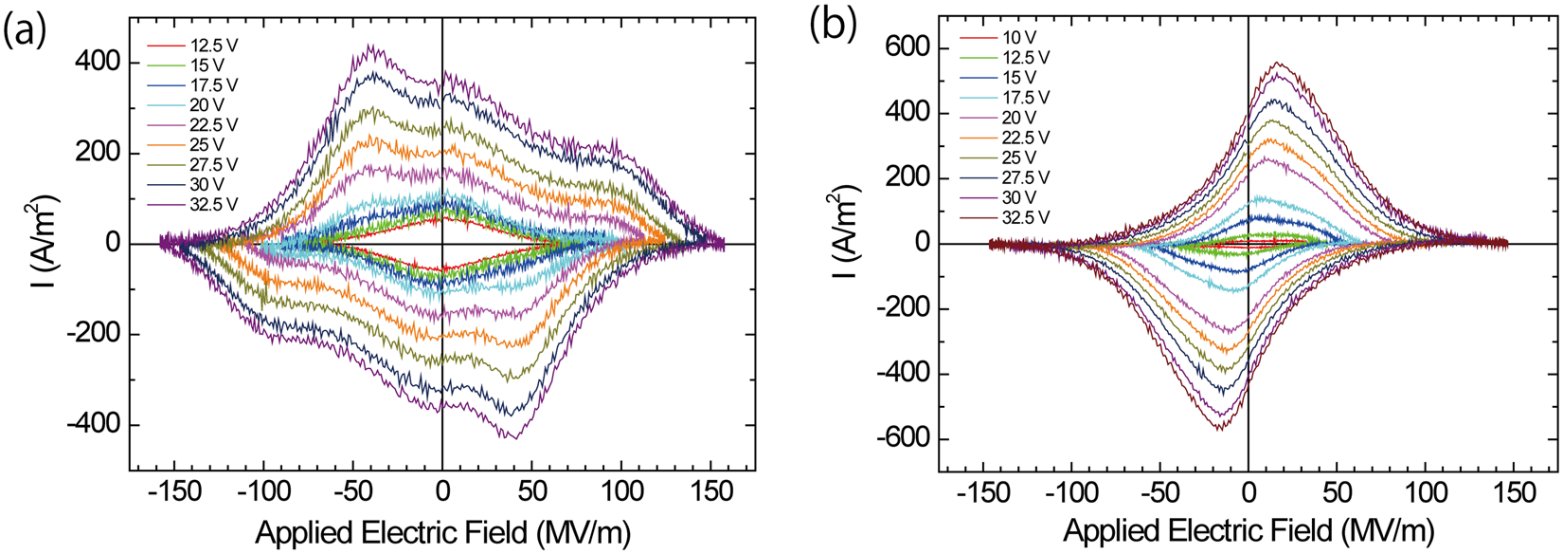
I-E loop after applying a sinusoidal electric field to the sample film with various maximum amplitudes and a 1k Hz cycle for the (**a**) P(VDF-TrFE-CFE) terpolymer and (**b**) P(VDF-TrFE-CTFE) terpolymer. Rhombus- or diamond-shaped loops at the lower maximum electric field strengths and more complexed loops with the increasing maximum electric field strength for the P(VDF-TrFE-CFE) terpolymer. (**b**) Rhombus- or diamond-shaped loop for P(VDF-TrFE-CTFE). For both cases, with an increasing maximum amplitude, the switching current increased. Thickness: 223.2 nm for P(VDF-TrFE-CFE) and 223.9 nm for P(VDF-TrFE-CTFE).

**Figure 3:**
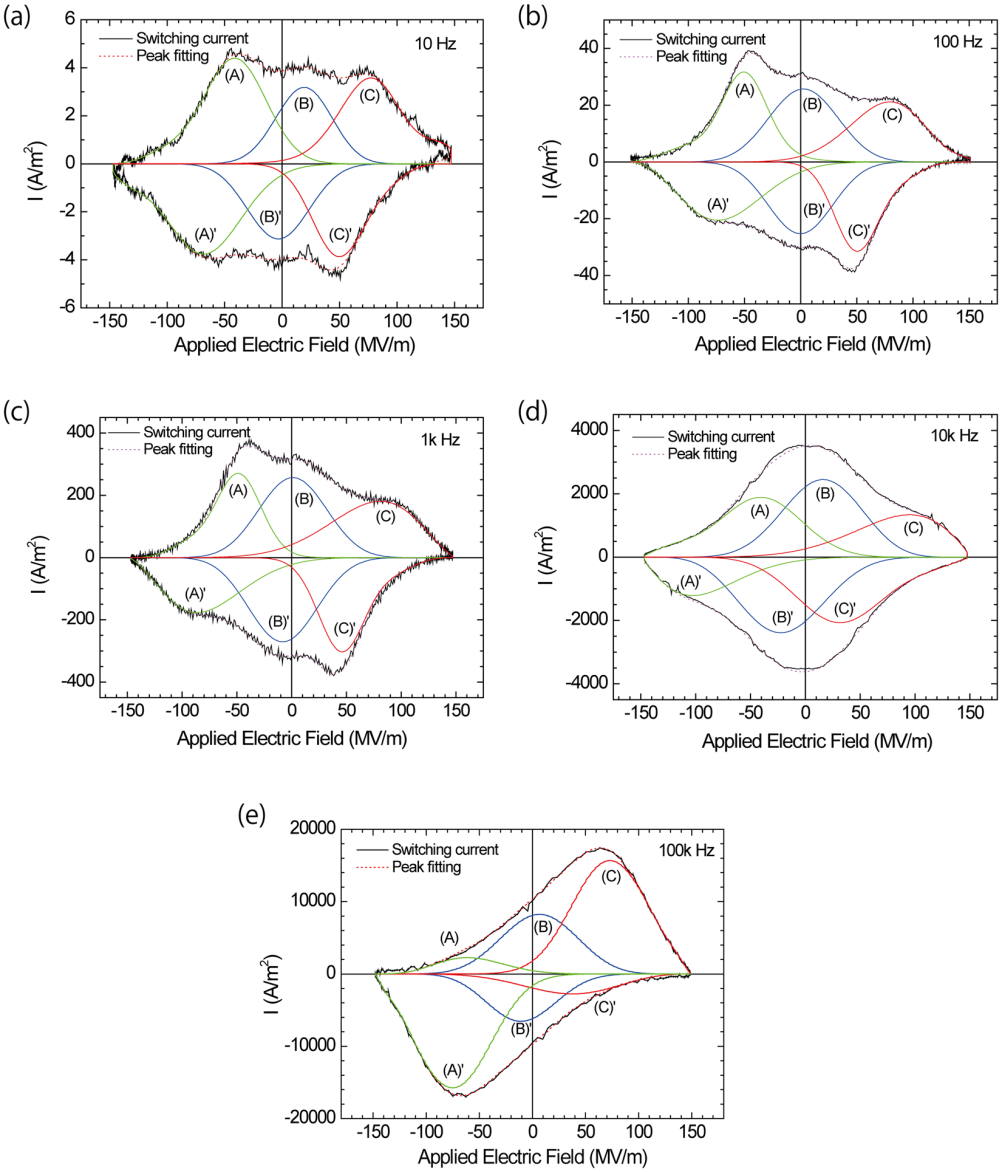
Switching current loops at various cycle frequencies and peak fitting using Gaussian peak currents. The positive current was reproduced using the summation of peaks (A) – (C). The negative current was reproduced using the summation of peaks (A)’ – (C)’. The reproduced current loop is expressed by the red dotted line in each figure. The applied electric field frequencies were (**a**) 10 Hz, (**b**) 100 Hz, (**c**) 1k Hz, (**d**) 10k Hz and (**e**) 100k Hz.

The switching current loops were measured by applying a sinusoidal electric field with various maximum electric field strengths, and they are shown in Fig. 2(a) for P(VDF-TrFE-CFE) and (b) for P(VDF-TrFE-CTFE). For P(VDF-TrFE-CFE), a rhombus- or diamond-shaped loop was observed when a lower maximum electric field was applied to the sample film. With increasing the maximum electric field, the current loop exhibited a complex shape. What is the origin for the switching behaviour between the lower electric field and higher electric field? We attributed the complex switching current loop to the existence of different switching dipoles. The complex switching current loop in the P(VDF-TrFE-CFE) terpolymer should have been the summation of several Gaussian peaks. Hence, a peak separation technique was employed to clarify the origin of the complex switching current loop in P(VDF-TrFE-CFE). The observed complex switching current – applied electric field (I-E) loop was reproduced using a summation of several Gaussian peaks reproduced using PeakFit^TM^ Ver. 4.0 software. The observed I-E loops at 10, 100, 1k, 10k, and 100k Hz are shown in Fig. 3. The I-E loop significantly depended on the switching frequency. Each I-E loop was independently reproduced using the summation of several Gaussian peaks, which are shown in the dashed curves in Fig. 3. The positive current was reproduced using the summation of peaks (A) – (C) at 10, 100 Hz, 1k, 10k, and 100k Hz. The negative current was reproduced using the summation of peaks (A)’ – (C)’ at 10, 100 Hz, 1k, 10k, and 100k Hz. Peaks (B) and (B)’ formed one pair of switching peaks at approximately 0 MV/m in the low electric field region. For peaks of (A), (A)’, (C) and (C)’, two models of pairing for switching were assumed: model (1) includes peak pairs of (A) and (C)’ and (A)’ and (C), and model (2) includes peak pairs of (A) and (A)’ and (C) and (C)’. The peak pair of (C) and (A)’ in model (1) represents well-known ferroelectric switching in P(VDF-TrFE), and the peak pair of (A) and (C)’ represents reversed switching. In model (2), the peak pair of (A) and (A)’ represents switching in the negative electric field region, and the peak pair of (C) and (C)’ represents switching in the positive electric field region. These switching behaviours should be the origin of the double hysteresis loop.

**Figure 4:**
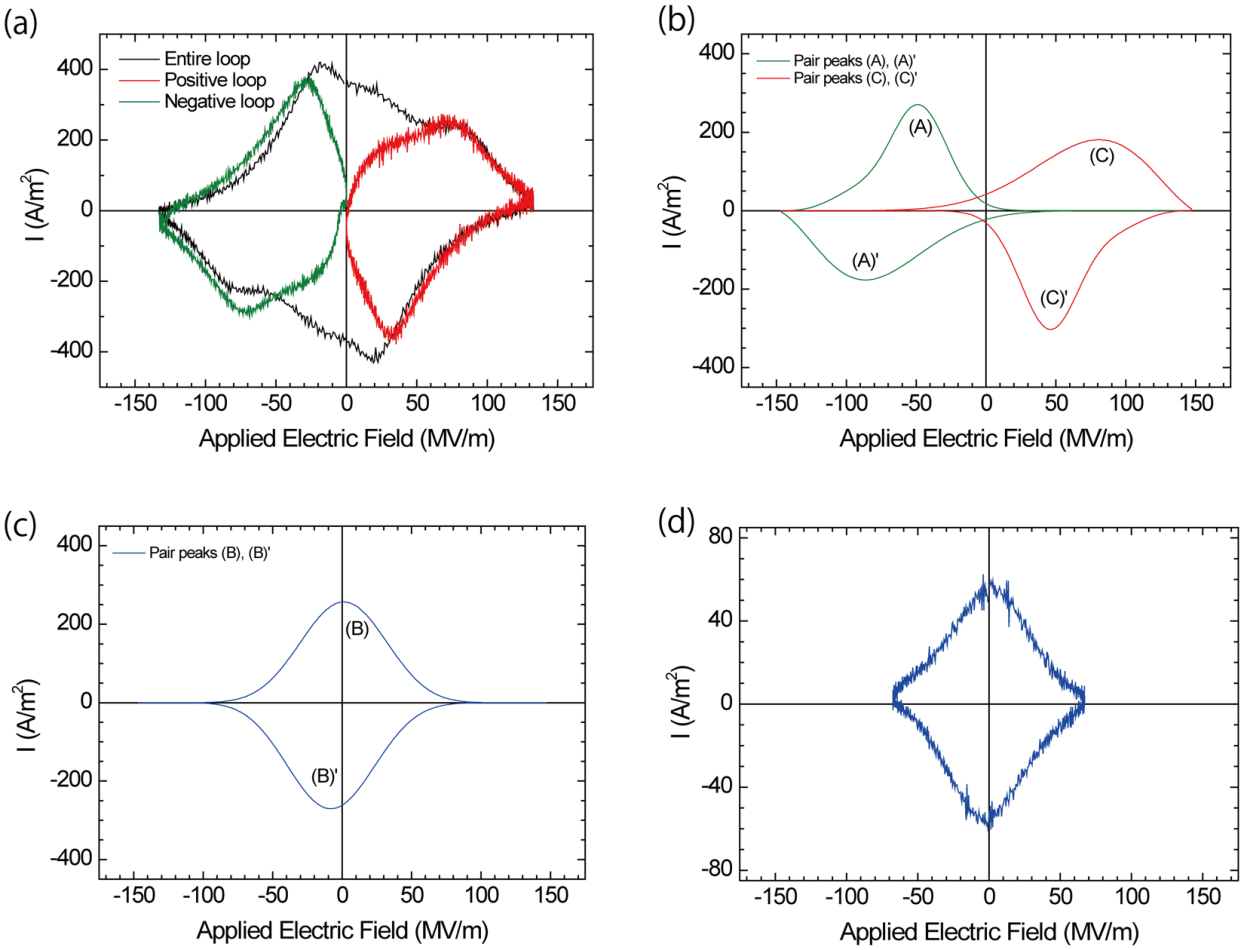
(**a**,**b**) Switching current loops under positive or negative electric fields and (**c**), (**d**) switching current loops under a series of positive and negative electric fields with a 1k Hz cycle. (**a**) Observed switching current loop: The green line is the switching current loop in the negative electric field region (−150 MV/m ≤ *E* ≤ 0). The red line is the switching current loop in the positive electric field region (0 ≤ *E* ≤ 150 MV/m). The black line is the normal switching current loop (−150 MV/m ≤ *E* ≤ 150 MV/m) as a reference. (**b**) Corresponding peak pair (A) and (A)’ for the negative electric field region and peak pair (C) and (C)’ for the positive electric field region from model (2). The observed switching current loop in (**a**) provides evidence of the double switching loop (double hysteresis loop). (**c**) Peak pair (B) and (B)’. (**d**) Observed switching current at the lower applied maximum electric field (−70 MV/m ≤ E ≤ 70 MV/m).

To confirm if the switching models were accurate, we measured switching current loops while applying positive or negative electric fields to the sample films at 1k Hz of cycling. This experimental approach is very important determining the origins of complex switching current loops. When only a positive or negative electric field was applied to the P(VDF-TrFE) copolymer thin film, no ferroelectric switching response (no switching current peak) was observed. This behaviour is natural for the P(VDF-TrFE) copolymer because series of positive and negative electric fields is needed to induce polarization reversal of the ferroelectric dipoles. Figure 4(a) shows the current loop of the P(VDF-TrFE-CFE) terpolymer when only a positive or negative electric field was applied. In Figure 4(a), current loops were observed when a positive field (red) of 0 ≤ *E* ≤ 150 MV/m and negative field (green) of −150 MV/m ≤ *E* ≤ 0 was applied. The current loop (black) corresponding to original switching was observed in the field range of −150 MV/m ≤ *E* ≤ 150 MV/m. Figure 4(b) displays the corresponding pair peaks (A) and (A)’ and (C) and (C)’ from model (2). The current loop (red) for the positive field of 0 ≤ *E* ≤ 150 MV/m corresponds to the peak pair (C) and (C)’ from model (2), and the current loop (green) for the negative field of −150 MV/m ≤ *E* ≤ 0 corresponds to the peak pair (A) and (A)’ from model (2). The results in Fig. 4(a) and (b) provide evidence that P(VDF-TrFE-CFE) exhibits double hysteresis or antiferroelectric-like switching. At high frequency cycling above 100k Hz, as shown in Fig. 3(e), the double hysteresis components appeared to be smaller, thus antiferroelectric to ferroelectric transitions may have occurred.

We discuss the assignment of the peak pair (B) and (B)’ in Fig. 4(c) and (d). The peak pair of (B) and (B)’ shown in Fig. 4(c) reasonably corresponds to the rhombus- or diamond-shaped loop observed when a lower maximum electric field (−70 MV/m ≤ *E* ≤ 70 MV/m) was applied to the sample film, as shown in Fig. 4(d).

The complex switching current loop for P(VDF-TrFE-CFE) was the summation of three different switching loops: the single switching loop in the negative electric field region (peak pair (A) and (A)’), that in the positive electric field region (peak pair (C) and (C)’), and that from the switching series of negative and positive electric fields (peak pair (B) and (B)’).

**Figure 5:**
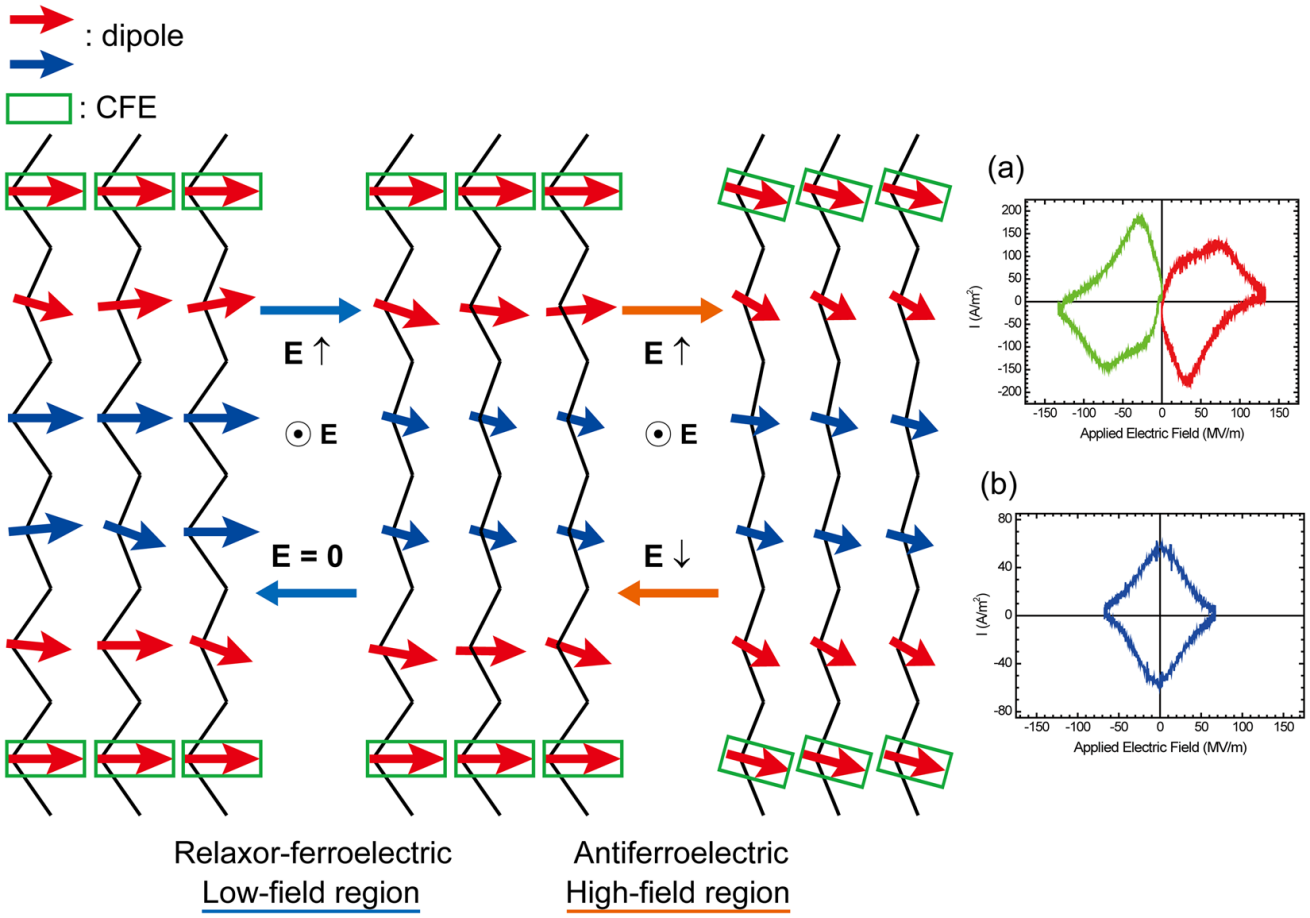
Schematic model of the dipole switching in the P(VDF-TrFE-CFE) terpolymer. CFE sites act as pinning sites. The VDF and TrFE dipoles neighbouring the CFE sites are tightly pinned by the CFE sites, as indicated by the red arrows. They can rotate at higher electric fields but are pulled back by the pinning sites upon reducing or removing the electric field. This switching behaviour caused the double hysteresis loop-like antiferroelectricity. The corresponding switching current is shown in (**a**). The rotating nanodomains of the VDF and TrFE dipoles far from the CFE sites, indicated by the blue arrows, enable switching, which led to relaxor ferroelectricity at lower electric field cycling. The corresponding switching current is shown in (**b**).

Based on the proposed switching model for relaxor-ferroelectric vinylidene fluoride terpolymers^10,11^, the switching mechanism for P(VDF-TrFE-CFE) terpolymers is presented. A physical pinning mechanism was proposed to explain the relaxor ferroelectricity in vinylidene fluoride terpolymers^10^. A schematic model of the dipole switching process is displayed in Fig. 5. The key part of the mechanism is the introduction of the third comonomer with bulky CFE sites. Third comonomer widened the polymer interchain distance, which was confirmed from the crystal lattice spacings using WAXD measurements. However, the rotation of the long-ranged polymer chains was restricted and pinned between neighbouring comonomers by introducing the third comonomer with bulky CFE sites^11^, which is illustrated in the schematic model of dipole switching in Fig. 5. In this case, the polymer chains neighbouring the CFE comonomers, which are indicated by the red arrows in Fig. 5, hardly rotate at lower positive electric fields, whereas at higher positive electric fields they can rotate but are pulled back by the pinning sites upon removal of the electric field. When applying a negative electric field, reversed switching occurs. These switching leads to a double hysteresis loop. These results can reasonably explain the switching currents in Fig. 4, and these currents are the origin of the double hysteresis loop in the P(VDF-TrFE-CFE) terpolymer.

We sought to understand the origin of the observed rhombus- or diamond-shaped current loop and peak pair (B) and (B)’ in Fig. 4(c) when lower maximum electric fields were applied to the sample film. This switching was attributed to the so-called relaxor-ferroelectric behaviour. This current loop only occurred when a series of positive and negative electric fields was applied. Hence, the origin of the polarization reversal was the same as that for the common ferroelectric loop that occurred in the PVDF and P(VDF-TrFE) copolymers, but the domain size of the rotating dipoles was smaller than that of PVDF and P(VDF-TrFE). The wider lattice spacing induced by the bulky comonomer CFE sites enabled the nanodomains of VDF and the TrFE sites to rotate far from the bulky CFE sites, indicated by the blue arrows in Fig. 5, at lower applied electric fields.

## Conclusion

In conclusion, we conducted switching current measurements to characterize the ferroelectricity of P(VDF-TrFE-CFE) and P(VDF-TrFE-CTFE) terpolymers. The switching current measurements definitely indicated the presence of relaxor ferroelectricity at lower electric field cycling and double hysteresis loop-like antiferroelectricity at higher electric field cycling. The pinning effect due to CFE restricted the rotation of the VDF and TrFE dipoles neighbouring the CFE sites, which caused double hysteresis switching. In contrast, the nanodomains of VDF and the TrFE sites far from the CFE site could still rotate, which caused relaxor ferroelectricity. This article presents a more comprehensive understanding of the novel ferroelectric behaviour of relaxor ferroelectricity and double hysteresis switching in VDF terpolymers, which will be directly connected to the development of devices for nonvolatile memory, electrostriction, electric energy storage, and electrocaloric cooling.

## Materials and Methods

Terpolymers of P(VDF-TrFE-CFE) (Piezotech, France) with the composition of 59/33/8 and terpolymers of P(VDF-TrFE-CTFE) (Piezotech, France) with the composition of 64.2/27.1/8.7 were supplied from Arkema, Japan. Copolymers of VDF and TrFE (P(VDF-TrFE)) with compositions of 75/25 were purchased from Kureha, Japan. Methyl ether ketone (MEK, Nakalai Tesque, Japan) was used as the solvent.

Each polymer was dissolved in MEK to prepare a 3 wt% polymer MEK solution. The polymer MEK solution was spin coated at 1000 rpm for 30 s onto a 5 mm *φ* gold electrode that was evaporated onto a silicon substrate. The obtained spin-coated thin film was dried under ambient conditions for 24 h, followed by thermal annealing for 2 h at 110 °C for the terpolymers of P(VDF-TrFE-CFE) and P(VDF-TrFE-CTFE) and for 2 h at 135 °C for the copolymer of P(VDF-TrFE). Another small top gold electrode was evaporated onto an annealed polymer thin film using a 117 μm × 117 μm mesh mask.

The thicknesses of sample films were measured using an atomic force microscope (AFM, Pacific Technology Nano-R, USA). The film thicknesses of the sample films were in the range of 200–260 nm. Wide angle X-ray diffraction (WAXD) patterns of the sample films were recorded using an X-ray diffractometer (Rigaku RINT2500, Japan). The thermal properties, the Curie temperature and melting point, were measured using a differential scanning calorimeter (DSC, TA Instruments Q2000).

The ferroelectric switching was measured using an FCE-1/1A ferroelectric measurement system (Toyo Corporation, Japan) in combination with a Nano-R AFM and a conductive diamond probe. Details for these measurements are described in previous reports^8,15,16^.

The switching current *J*(t) is obtained from the following equation

1 $$J\,(t)=\sum _{j}\frac{d{P}_{j}}{dt}+\varepsilon {\varepsilon }_{0}\frac{dE}{dt}+\frac{E}{\rho }$$

when the sample film was subjected to a cyclic applied electric field. Here, *P* is the polarization, *ε* is the relative dielectric constant, *ε*_0_ is the permeability in vacuum, *ρ* is the resistivity, and *E* is the applied electric field. The relative dielectric constant of the sample film was evaluated using the capacitance term parallel to dE/dt. After integrating the current and subtracting the capacitance and resistivity terms, the polarization as a function of the applied electric field (hysteresis loop) was obtained, from which the remanent polarization (*P*_r_) and cohesive electric field (*E*_c_) were evaluated.
